# 5,5-Di­chloro-6-hy­droxy­dihydro­pyrimidine-2,4(1*H*,3*H*)-dione: mol­ecular and crystal structure, Hirshfeld surface analysis and the new route for synthesis with Mg(ReO_4_)_2_ as a new catalyst

**DOI:** 10.1107/S2056989020011809

**Published:** 2020-09-04

**Authors:** Anton P. Novikov, Sergey N. Ryagin, Mikhail S. Grigoriev, Alexey V. Safonov, Konstantin E. German

**Affiliations:** a Peoples’ Friendship University of Russia, 6 Miklukho-Maklaya St, 117198, Moscow, Russian Federation; b A. N. Frumkin Institute of Physical Chemistry and Electrochemistry, Russian Academy of Sciences, 31 Leninsky Prospekt bldg 4, 119071 Moscow, Russian Federation; c Medical University REAVIZ, Moscow branch, Krasnobogatyrskaya 2, 107564 Moscow, Russian Federation

**Keywords:** crystal structure, uracil, pyrimidine, hydrogen bonds, halogen bonds, Hirshfeld surface analysis

## Abstract

The mol­ecular and crystal structures of the title compound, C_4_H_4_Cl_2_N_2_O_3_, were investigated by single-crystal X-ray diffraction and a Hirshfeld surface analysis. The title compound was synthesized by a new type of reaction using Mg(ReO_4_)_2_ as a new catalyst and a possible mechanism for this reaction is proposed. The six-membered ring adopts a half-chair conformation. In the crystal, hydrogen bonds connect the mol­ecules into double layers, which are connected to each other by halogen bonds. The Hirshfeld surface analysis revealed that the most important contributions for the crystal packing are from O⋯H/H⋯O (35.8%), Cl⋯Cl (19.6%), Cl⋯H/H⋯Cl (17.0%), H⋯H (8.3%), C⋯O/O⋯C (4.3%), Cl⋯O/O⋯Cl (4.2%) and O⋯O (4.1%) contacts.

## Abstract 2

The title compound was synthesized by a new type of reaction using Mg(ReO_4_)_2_ as a new catalyst and a possible mechanism for this reaction is proposed. In the crystal, hydrogen bonds connect the mol­ecules into double layers, which are connected to each other by halogen bonds.

## Chemical context   

Nitro­gen heterocycles and pyrimidines are examples of the most important biologically active compounds and find a wide use in modern medicine (Pałasz *et al.*, 2015 ▸; Takeshita *et al.*, 2006 ▸; Henderson *et al.*, 2003 ▸). Uracil is widespread in nature as a pyrimidine derivative, and is found as a part of nucleic acids. Uracil derivatives are used for therapeutic purposes (Smith *et al.*, 2004 ▸; Kasradze *et al.*, 2012 ▸) . Halogen derivatives of uracil serve as convenient inter­mediates for the preparation of compounds with various functional groups (Wamhoff *et al.*, 1992 ▸). Halogen–halogen bonding has recently attracted attention as it expands the possibilities of understanding the new properties of compounds containing halogens and their applications (Szell *et al.*, 2017 ▸). Pyrimidine derivatives are used as inter­mediates for the production of various complex organic mol­ecules for the treatment of cancer and AIDS (Fawcett *et al.*, 1996 ▸). Several pyrimidine derivatives belong to the class of central nervous system depressants (Soayed *et al.*, 2015 ▸).
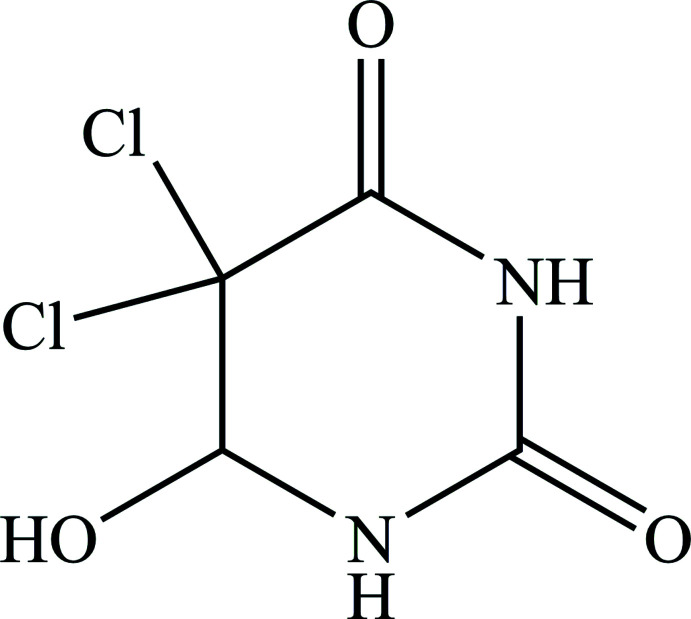


**Figure 1:**
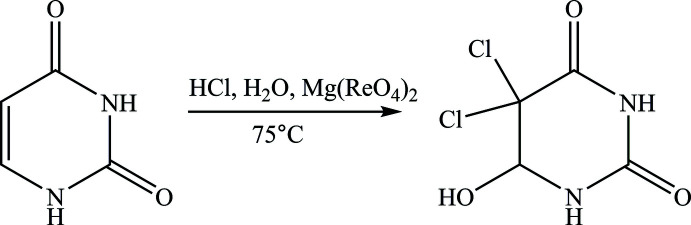
Synthesis scheme of **1.**

5,5-Di­chloro-6-hy­droxy­dihydro­pyrimidine-2,4(1*H*,3*H*)-di­one (**1**) was earlier synthesized by two reaction schemes starting with uracil: (1) by addition of Cl_2_ in H_2_O (Johnson *et al.*, 1943 ▸) or (2) by addition of Na_2_S_2_O_8_ and NaCl in acetic acid (Itahara *et al.*, 1986 ▸). We have found a new reaction for the synthesis of **1** by the reaction of uracil with hydro­chloric acid and water in the presence of Mg(ReO_4_)_2_ as a catalyst. The reaction scheme is shown in Fig. 1 ▸.

## Structural commentary   

**Figure 2:**
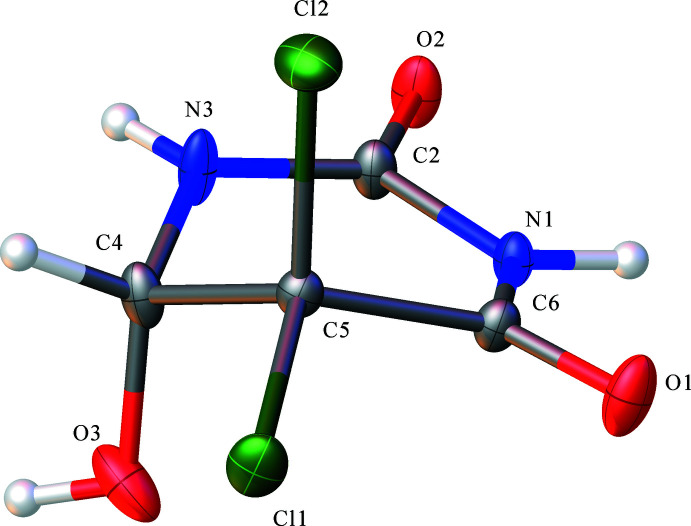
Mol­ecular structure of the title compound, including atom labelling. Displacement ellipsoids are drawn at the 50% probability level.

The title compound crystallizes in the space group *C*2/*c* with eight mol­ecules in the unit cell. The asymmetric unit is illustrated in Fig. 2 ▸. A similar compound with a methyl group instead of an H atom at C5 (ZEQYIF; Kasradze *et al.*, 2012 ▸; ZEQYIF01; Sharutin, 2016 ▸) crystallizes in the space group *P* 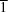. The six-membered ring adopts a half-chair conformation, as in ZEQYIF (Kasradze *et al.*, 2012 ▸). The largest angle at nitro­gen atom, C6—N1—C2, is 126.69 (13)°. The angle involving the two chlorine atoms, Cl1—C5—Cl2, is 109.29 (8)°. The two C—N bonds at the N1 atom are similar in length while those at N3 differ because of the N3—C2 *sp* ^2^-conjugation, the latter bond being only 1.344 (2) Å (Table 1 ▸). The six atoms N3, C2, O2, N1, C6 and O1 are almost planar (r.m.s. deviation of fitted atoms = 0.0462 Å) while the two other ring atoms of the ring C4 and C5 are displaced from this plane by −0.275 (2) and 0.411 (3) Å, respectively, forming the above mentioned half-chair.

## Supra­molecular features   

**Figure 3:**
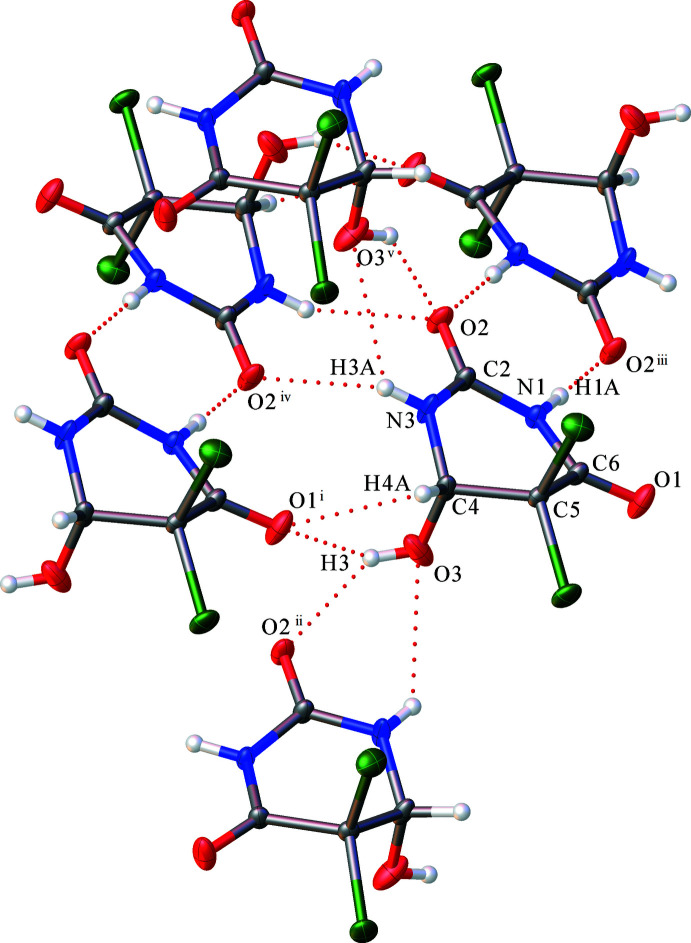
View showing the hydrogen bonds in **1**. [Symmetry codes: (i) *x*, *y* + 1, *z*; (ii) *x*, −*y* + 2, *z* + 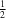; (iii) −*x*, −*y* + 1, −*z* + 1; (iv) −*x*, −*y* + 2, −*z* + 1; (v) *x*, −*y* + 2, *z* − 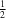.]

**Figure 4:**
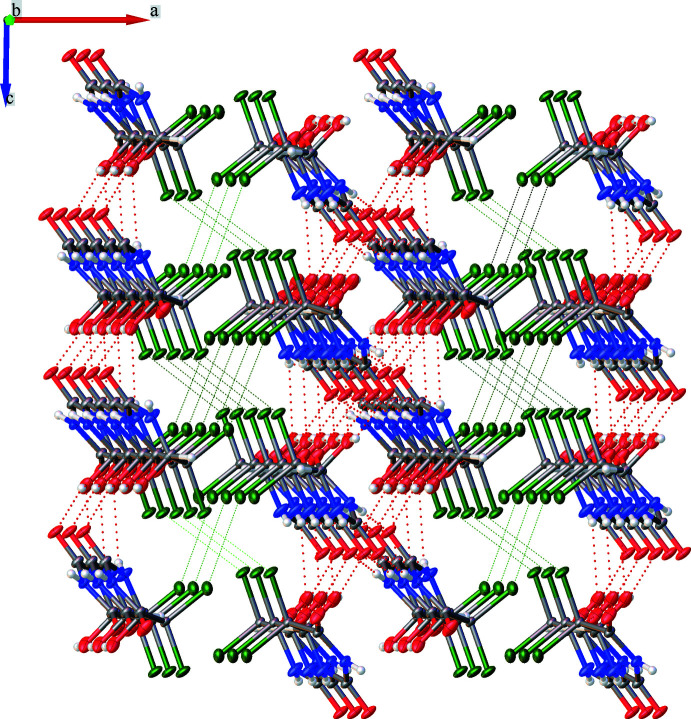
Crystal packing of **1** showing the double layers with halogen bonds between them.

The hydrogen-bond system is shown in Fig. 3 ▸. In the structure, there are two bifurcated hydrogen bonds. O3—H3 forms a bifurcated hydrogen bond with the O1^i^ and O2^ii^ atoms [symmetry codes: (i) *x*, *y* + 1, *z;* (ii) *x*, −*y* + 2, *z* + 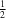] of different mol­ecules. The C4—H4*A*⋯O1^i^ hydrogen bond also involves O1^i^ with a H4*A*⋯O1^i^ distance of 3.058 (2) Å (Table 2 ▸). In the similar compound ZEQYIF (Kasradze *et al.*, 2012 ▸), the OH group participates as proton donor in a very strong hydrogen bond with the O atom of one of the CO groups of a neighbouring mol­ecule. In our crystal structure, such strong hydrogen bonds are absent. N3—H3*A* forms a bifurcated hydrogen bond weaker than O3—H3 with atoms O2^iv^ and O3^v^ [symmetry codes: (iv) −*x*, −*y* + 2, −*z* + 1; (v) *x*, −*y* + 2, *z* − 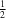] of different mol­ecules. The strongest hydrogen bond is N1—H1*A*⋯O2^iii^ [symmetry code: (iii) −*x*, −*y* + 1, −*z* + 1] with an N1⋯O2^iii^ distance of 2.793 (2) Å. The hydrogen bonds connect the mol­ecules into double layers parallel to the (100) plane, as shown in Fig. 4 ▸. Halogen bonds Cl1⋯Cl1^vi^ [3.3670 (9) Å] and Cl2⋯Cl2^vii^ [3.3568 (8) Å; symmetry codes: (vi) 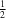 − *x*, 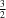 − *y*, 2 − *z*; (vii) 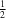 − *x*, 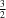 − *y*, 1 − *z*] connect the layers, forming a three-dimensional framework.

## Hirshfeld surface analysis   

**Figure 5:**
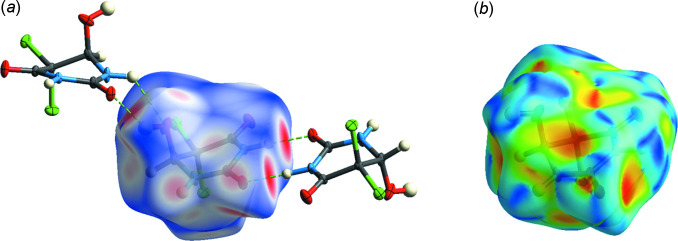
Hirshfeld surface mapper over (*a*) *d*_norm_ and (*b*) shape-index to visualize the inter­actions in the title compound.

**Figure 6:**
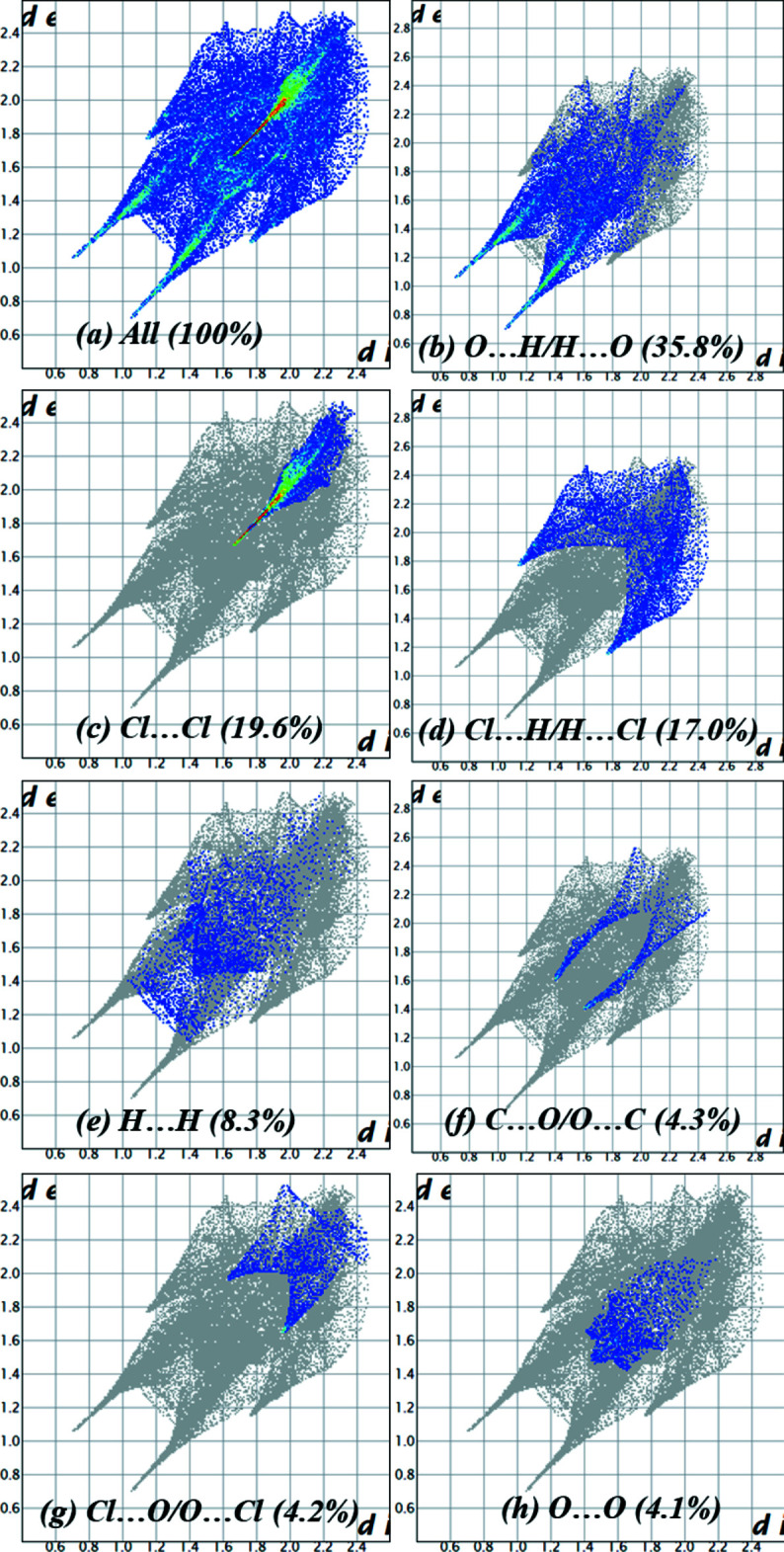
(*a*) A full two-dimensional fingerprint plot for the title compound, together with those delineated into (*b*) O⋯H/H⋯O, (*c*) Cl⋯Cl, (*d*) Cl⋯H/H⋯Cl, (*e*) H⋯H, (*f*) C⋯O/O⋯C, (*g*) Cl⋯O/O⋯Cl and (*h*) O⋯O contacts.

The *Crystal Explorer 17.5* (Turner *et al.*, 2017 ▸) program was used to analyse the inter­actions within the crystal. The donor–acceptor groups are visualized using a standard (high) surface resolution and *d*_norm_ surfaces are mapped over a fixed colour scale of −0.640 (red) to 0.986 (blue) a.u., as illustrated in Fig. 5 ▸. Red spots on the surface of the *d*_norm_ plot indicate inter­molecular contacts involving the hydrogen and halogen bonds. The brightest red spots correspond to the strongest hydrogen bonds, N1—H1*A*⋯O2 and O3—H3⋯O2 (Table 2 ▸). There are no π–π inter­actions in the mol­ecule, as can be seen from Fig. 5 ▸ *b* by the absence of characteristic triangles. The fingerprint plots (Fig. 6 ▸) show that the O⋯H/H⋯O contacts (35.8%) make the largest contribution to the overall packing of the crystal, which is due to the fact that hydrogen bonds of the O—H⋯O and N—H⋯O types are predominantly formed in the crystal. Then, the Cl⋯Cl (19.6%) and Cl⋯H/H⋯Cl (17.0%) contacts make approximately the same contribution. H⋯H (8.3%) contacts make an insignificant contribution, similarly for the C⋯O/O⋯C (4.3%), Cl⋯O/O⋯Cl (4.2%) and O⋯O (4.1%) contacts, which make approximately the same contribution. Other contacts make weaker contributions to the packaging and are not shown in Fig. 6 ▸.

## Database survey   

A search of the Cambridge Structural Database (CSD, Version 5.41, update of November 2019; Groom *et al.*, 2016 ▸) for different possible substituents at C4 and C5 atoms gave only a few results. A similar compound was found with a methyl group instead of an H atom at C5 (ZEQYIF; Kasradze *et al.*, 2012 ▸; ZEQYIF01; Sharutin, 2016 ▸). In *cis*-thymine glycol (THYGLY10; Flippen, 1973 ▸), one of the chlorine atoms is replaced by an OH group, and the second chlorine atom is replaced by a methyl group. FUFDIT (Flippen-Anderson, 1987 ▸) is the same as *cis*-thymine glycol, except that the H atom in the hydroxyl group is replaced by OH group and water of crystallization is present.

## Synthesis and crystallization   

The title compound was synthesized by adding 5 mg of uracil (Sigma Aldrich) to 1 ml of 1 mol l^−1^ hydro­chloric acid aqueous solution in the presence of magnesium perrhenate. This solution was heated in a water bath (at 348 K) until the components were completely dissolved. Crystallization occurred with isothermal evaporation of the resulting solution at room temperature for several weeks, giving colourless needle-shaped crystals, composition according to chemical analysis (obs./calc.): C, 24.12/24.14; H, 2.04/2.03; Cl, 35.64/35.63; N, 14.07/14.08; O, 24.13/24.12. Crystals suitable for a X-ray structural analysis were extracted manually from this batch.

We suggest a possible mechanism of the observed reaction. Typically, ReO_4_^−^ does not react with HCl while TcO_4_^−^ is actively reduced (German *et al.*, 2002 ▸). We found that in the presence of Mg^2+^, the ReO_4_^−^ being distorted according to (Khrustalev, 2000 ▸; Ravi *et al.*, 2018 ▸) attacks the HCl, forming Cl_2_ that is readily reacted with water to form HOCl. In the air and in low acidic HCl·H_2_O solution, the Re is then oxidized back to Re^VII^. Cl_2_ is thus formed by the action of hydro­chloric acid on the rhenium salt as a result of a redox reaction. The process of hypohalogenation is then likely to occur. Since the reaction takes place in an aqueous medium, the formation of hypohalogenic acid is possible by the reaction Cl_2_ + H_2_O = HOCl + HCl. Hypohalogenation is usually carried out with an aqueous solution of halogen. The addition to positions 5 and 6 is electrophilic, in accordance with the electron-density distribution. The partially positively charged halogen atom is directed towards carbon C5, which has a higher partial negative charge compared to the C6 atom, towards which the hydroxyl is directed. Then, at position C5, hydrogen is possibly replaced by Cl_2_ by electrophilic (more likely in an aqueous medium) and possibly through radical substitution.

## Refinement   

Crystal data, data collection and structure refinement details are summarized in Table 3 ▸. The C-bound hydrogen atom was placed at a calculated position (C—H = 1.00 Å) and refined using a riding-atom model [*U*_iso_(H) = 1.2*U*_eq_(C)]. O- and N-bound H atoms were refined isotropically [*U*_iso_(H) = 1.2*U*_eq_(O, N)].

## Supplementary Material

Crystal structure: contains datablock(s) I, global. DOI: 10.1107/S2056989020011809/zq2257sup1.cif

Structure factors: contains datablock(s) I. DOI: 10.1107/S2056989020011809/zq2257Isup2.hkl

Supporting information file. DOI: 10.1107/S2056989020011809/zq2257Isup3.cml

CCDC reference: 2025758

Additional supporting information:  crystallographic information; 3D view; checkCIF report

## Figures and Tables

**Table 1:** Selected geometric parameters (Å, °)

N1—C6	1.3658 (19)	N3—C4	1.447 (2)
N1—C2	1.375 (2)	C4—C5	1.531 (2)
N3—C2	1.344 (2)	C5—C6	1.536 (2)
			
C6—N1—C2	126.69 (13)	Cl1—C5—Cl2	109.29 (8)

**Table 2:** Hydrogen-bond geometry (Å, °)

*D*—H⋯*A*	*D*—H	H⋯*A*	*D*⋯*A*	*D*—H⋯*A*
O3—H3⋯O1^i^	0.81 (2)	2.41 (2)	3.045 (2)	136 (2)
O3—H3⋯O2^ii^	0.81 (2)	2.16 (2)	2.8076 (18)	138 (2)
N1—H1*A*⋯O2^iii^	0.89 (2)	1.91 (2)	2.7932 (17)	178 (2)
N3—H3*A*⋯O2^iv^	0.79 (2)	2.46 (2)	3.0978 (19)	138 (2)
N3—H3*A*⋯O3^v^	0.79 (2)	2.60 (2)	3.147 (2)	128 (2)
C4—H4*A*⋯O1^i^	1.00	2.45	3.058 (2)	119

Symmetry codes: (i) 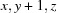; (ii) 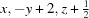; (iii) 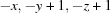; (iv) 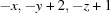; (v) 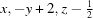.

**Table 3:** Experimental details

Crystal data	
Chemical formula	C_4_H_4_Cl_2_N_2_O_3_
*M*_r_	198.99
Crystal system, space group	Monoclinic, *C*2/*c*
Temperature (K)	100
*a*, *b*, *c* (Å)	19.9042 (10), 6.6243 (4), 10.5636 (7)
β (°)	90.819 (4)
*V* (Å^3^)	1392.68 (14)
*Z*	8
Radiation type	Mo *K*α
μ (mm^−1^)	0.89
Crystal size (mm)	0.50 × 0.10 × 0.02

Data collection	
Diffractometer	Bruker Kappa APEXII area-detector diffractometer
Absorption correction	Multi-scan (*SADABS*; Bruker, 2008 ▸)
*T*_min_, *T*_max_	0.802, 0.983
No. of measured, independent and observed [*I* > 2σ(*I*)] reflections	12430, 3046, 2128
*R*_int_	0.045
(sin θ/λ)_max_ (Å^−1^)	0.807

Refinement	
*R*[*F* ^2^ > 2σ(*F* ^2^)], *wR*(*F* ^2^), *S*	0.042, 0.103, 1.02
No. of reflections	3046
No. of parameters	109
No. of restraints	3
H-atom treatment	H atoms treated by a mixture of independent and constrained refinement
Δρ_max_, Δρ_min_ (e Å^−3^)	0.91, −0.55

Computer programs: *APEX2* and *SAINT-Plus* (Bruker, 2012 ▸), *SHELXS97* (Sheldrick, 2008 ▸) and *SHELXL2018* (Sheldrick, 2015 ▸).

## supplementary crystallographic information

### Crystal data

**Table:**

C_4_H_4_Cl_2_N_2_O_3_	*F*(000) = 800
*M**_r_* = 198.99	*D*_x_ = 1.898 Mg m^−^^3^
Monoclinic, *C*2/*c*	Mo *K*α radiation, λ = 0.71073 Å
*a* = 19.9042 (10) Å	Cell parameters from 3314 reflections
*b* = 6.6243 (4) Å	θ = 3.8–34.7°
*c* = 10.5636 (7) Å	µ = 0.89 mm^−^^1^
β = 90.819 (4)°	*T* = 100 K
*V* = 1392.68 (14) Å^3^	Needle, colourless
*Z* = 8	0.50 × 0.10 × 0.02 mm

### Data collection

**Table:**

Bruker Kappa APEXII area-detector diffractometer	2128 reflections with *I* > 2σ(*I*)
φ and ω scans	*R*_int_ = 0.045
Absorption correction: multi-scan (SADABS; Bruker, 2008)	θ_max_ = 35.0°, θ_min_ = 4.1°
*T*_min_ = 0.802, *T*_max_ = 0.983	*h* = −32→31
12430 measured reflections	*k* = −9→10
3046 independent reflections	*l* = −17→16

### Refinement

**Table:**

Refinement on *F*^2^	3 restraints
Least-squares matrix: full	Hydrogen site location: mixed
*R*[*F*^2^ > 2σ(*F*^2^)] = 0.042	H atoms treated by a mixture of independent and constrained refinement
*wR*(*F*^2^) = 0.103	*w* = 1/[σ^2^(*F*_o_^2^) + (0.0468*P*)^2^ + 0.922*P*] where *P* = (*F*_o_^2^ + 2*F*_c_^2^)/3
*S* = 1.02	(Δ/σ)_max_ < 0.001
3046 reflections	Δρ_max_ = 0.91 e Å^−^^3^
109 parameters	Δρ_min_ = −0.55 e Å^−^^3^

### Special details

**Table:**

Geometry. All esds (except the esd in the dihedral angle between two l.s. planes) are estimated using the full covariance matrix. The cell esds are taken into account individually in the estimation of esds in distances, angles and torsion angles; correlations between esds in cell parameters are only used when they are defined by crystal symmetry. An approximate (isotropic) treatment of cell esds is used for estimating esds involving l.s. planes.

### Fractional atomic coordinates and isotropic or equivalent isotropic displacement parameters (Å^2^)

**Table:**

	*x*	*y*	*z*	*U*_iso_*/*U*_eq_	
Cl1	0.18354 (2)	0.69299 (6)	0.90632 (4)	0.02208 (10)	
Cl2	0.22188 (2)	0.68819 (7)	0.64394 (4)	0.02409 (11)	
O1	0.11148 (7)	0.36996 (19)	0.76917 (14)	0.0270 (3)	
O2	0.01449 (6)	0.75547 (19)	0.46558 (12)	0.0218 (3)	
O3	0.07140 (7)	0.9344 (2)	0.82106 (14)	0.0296 (3)	
H3	0.0619 (12)	1.052 (3)	0.828 (2)	0.036*	
N1	0.05958 (6)	0.57272 (19)	0.62634 (13)	0.0142 (2)	
H1A	0.0352 (10)	0.469 (3)	0.598 (2)	0.017*	
N3	0.08836 (7)	0.9086 (2)	0.60091 (15)	0.0209 (3)	
H3A	0.0803 (11)	1.010 (4)	0.564 (2)	0.025*	
C2	0.05203 (8)	0.7500 (2)	0.55987 (15)	0.0160 (3)	
C4	0.11860 (8)	0.9140 (2)	0.72628 (16)	0.0177 (3)	
H4A	0.152164	1.026060	0.731982	0.021*	
C5	0.15341 (7)	0.7110 (2)	0.74935 (15)	0.0135 (3)	
C6	0.10627 (7)	0.5334 (2)	0.71907 (15)	0.0148 (3)	

### Atomic displacement parameters (Å^2^)

**Table:**

	*U*^11^	*U*^22^	*U*^33^	*U*^12^	*U*^13^	*U*^23^
Cl1	0.02396 (18)	0.0258 (2)	0.01618 (18)	−0.00256 (15)	−0.01093 (14)	0.00345 (15)
Cl2	0.01459 (16)	0.0329 (2)	0.0248 (2)	−0.00212 (15)	0.00148 (14)	−0.00355 (17)
O1	0.0290 (6)	0.0180 (6)	0.0334 (8)	−0.0055 (5)	−0.0147 (5)	0.0107 (5)
O2	0.0269 (6)	0.0176 (5)	0.0204 (6)	−0.0034 (4)	−0.0153 (5)	0.0025 (5)
O3	0.0226 (6)	0.0338 (7)	0.0323 (8)	0.0093 (5)	−0.0071 (5)	−0.0185 (6)
N1	0.0149 (5)	0.0114 (5)	0.0160 (6)	−0.0017 (4)	−0.0059 (4)	0.0003 (5)
N3	0.0280 (7)	0.0099 (6)	0.0242 (8)	−0.0025 (5)	−0.0163 (6)	0.0042 (5)
C2	0.0174 (6)	0.0133 (6)	0.0169 (7)	−0.0003 (5)	−0.0075 (5)	0.0008 (5)
C4	0.0195 (7)	0.0133 (6)	0.0200 (7)	0.0019 (5)	−0.0104 (6)	−0.0028 (6)
C5	0.0122 (5)	0.0149 (6)	0.0132 (6)	0.0001 (5)	−0.0038 (5)	0.0013 (5)
C6	0.0147 (6)	0.0143 (6)	0.0152 (7)	−0.0011 (5)	−0.0048 (5)	0.0025 (5)

### Geometric parameters (Å, º)

**Table:**

Cl1—C5	1.7592 (15)	N1—H1A	0.89 (2)
Cl2—C5	1.7787 (16)	N3—C2	1.344 (2)
O1—C6	1.2086 (19)	N3—C4	1.447 (2)
O2—C2	1.2371 (18)	N3—H3A	0.79 (2)
O3—C4	1.389 (2)	C4—C5	1.531 (2)
O3—H3	0.805 (16)	C4—H4A	1.0000
N1—C6	1.3658 (19)	C5—C6	1.536 (2)
N1—C2	1.375 (2)		
			
C4—O3—H3	109.1 (19)	O3—C4—H4A	110.0
C6—N1—C2	126.69 (13)	N3—C4—H4A	110.0
C6—N1—H1A	117.2 (13)	C5—C4—H4A	110.0
C2—N1—H1A	115.5 (13)	C4—C5—C6	111.48 (12)
C2—N3—C4	121.97 (14)	C4—C5—Cl1	110.91 (11)
C2—N3—H3A	113.8 (17)	C6—C5—Cl1	110.09 (10)
C4—N3—H3A	120.2 (17)	C4—C5—Cl2	108.90 (11)
O2—C2—N3	123.56 (15)	C6—C5—Cl2	106.02 (11)
O2—C2—N1	119.77 (14)	Cl1—C5—Cl2	109.29 (8)
N3—C2—N1	116.65 (13)	O1—C6—N1	122.56 (14)
O3—C4—N3	112.65 (13)	O1—C6—C5	123.13 (13)
O3—C4—C5	106.26 (14)	N1—C6—C5	114.24 (13)
N3—C4—C5	107.77 (13)		
			
C4—N3—C2—O2	164.69 (16)	O3—C4—C5—Cl2	−172.67 (10)
C4—N3—C2—N1	−17.2 (2)	N3—C4—C5—Cl2	66.35 (14)
C6—N1—C2—O2	169.06 (16)	C2—N1—C6—O1	−176.62 (17)
C6—N1—C2—N3	−9.1 (3)	C2—N1—C6—C5	0.6 (2)
C2—N3—C4—O3	−70.5 (2)	C4—C5—C6—O1	−152.65 (17)
C2—N3—C4—C5	46.4 (2)	Cl1—C5—C6—O1	−29.1 (2)
O3—C4—C5—C6	70.70 (16)	Cl2—C5—C6—O1	88.98 (18)
N3—C4—C5—C6	−50.28 (18)	C4—C5—C6—N1	30.10 (19)
O3—C4—C5—Cl1	−52.36 (14)	Cl1—C5—C6—N1	153.63 (12)
N3—C4—C5—Cl1	−173.35 (11)	Cl2—C5—C6—N1	−88.27 (14)

### Hydrogen-bond geometry (Å, º)

**Table:**

*D*—H···*A*	*D*—H	H···*A*	*D*···*A*	*D*—H···*A*
O3—H3···O1^i^	0.81 (2)	2.41 (2)	3.045 (2)	136 (2)
O3—H3···O2^ii^	0.81 (2)	2.16 (2)	2.8076 (18)	138 (2)
N1—H1*A*···O2^iii^	0.89 (2)	1.91 (2)	2.7932 (17)	178 (2)
N3—H3*A*···O2^iv^	0.79 (2)	2.46 (2)	3.0978 (19)	138 (2)
N3—H3*A*···O3^v^	0.79 (2)	2.60 (2)	3.147 (2)	128 (2)
C4—H4*A*···O1^i^	1.00	2.45	3.058 (2)	119

Symmetry codes: (i) *x*, *y*+1, *z*; (ii) *x*, −*y*+2, *z*+1/2; (iii) −*x*, −*y*+1, −*z*+1; (iv) −*x*, −*y*+2, −*z*+1; (v) *x*, −*y*+2, *z*−1/2.
